# Moving Beyond the Binary: How Language and Common Research Practices Can Make Emergency Medicine Less Welcoming for Some Learners and Physicians

**DOI:** 10.5811/westjem.2022.8.58646

**Published:** 2022-10-24

**Authors:** Alex Farthing, John Burkhardt

**Affiliations:** * University of Pittsburgh Medical Center, Department of Emergency Medicine, Pittsburgh, Pennsylvania; † University of Michigan Medical School, Departments of Emergency Medicine and Learning Health Sciences, Ann Arbor, Michigan

Gender representation matters to our learners^1^,^2^ and our patients,^3^,^4^ but emergency physicians have historically been disproportionately white men.^5^ Despite an increase in the number of women medical students,^6^ emergency medicine (EM) still has fewer women applying to the specialty than would be expected from the overall number of medical graduates.^7^,^8^ The current state of representation of sexual and gender minority (SGM) physicians is less well described, but emergency medicine has not been reported as one of the more welcoming specialties for these learners, and the presence of SGM physicians was correlated with an increased culture of inclusion.^9^

In the current issue of *West*JEM, Gibney et al. report EM residency gender composition along purely binary lines. However, the composition of EM residents includes physicians who are outside of traditional binary definitions of sex and gender. A lack of acknowledgement of our colleagues from these backgrounds has important ramifications for them and our patients.

In a 2010 survey of SGM patients, most believed that providers were not adequately prepared to care for their needs.^10^ In that same study, more than half of the respondents reported facing discrimination when accessing health care, ranging from outright refusal of care to being subject to abusive language.^10^ Similarly, in a 2015 survey of transgender patients, nearly half reported avoiding the emergency department when they required acute care, citing fear of discrimination and previous negative experiences.^11^ An entire group of patients reporting such negative experiences when seeking care should be a clarion call for significant reform.

One potential solution to SGM patients’ significant discomfort in seeking emergency care would be diversifying the composition of emergency physicians to reflect the general population more closely. As racial diversity in medicine has increased, studies have shown that racial concordance between patient and provider can improve both patient satisfaction and participation in health care decisions.^4^,^12^ Similar benefits to SGM patient care may come from cultivating diversity in sexual and gender identity in medicine. There is already data suggesting that increased visibility of SGM providers is linked to a more welcoming environment for SGM patients.^13^ There is also evidence that many patients prefer to be treated by a doctor of a specific gender, which has implications for equity of access to care when gender diversity in medicine is limited.^4^ Data is limited, however, as few studies have explored gender diversity in medicine, and most large scale sources of data have only assessed gender in binary terms.

Gibney et al., explored the potential effect of having more women in positions of leadership in emergency medicine departments on the make-up of their residency classes. In their study, the authors used photographs to assign gender to residents and faculty members. We believe this approach provides an opportunity for reflection on how current research practices and normative behaviors in emergency medicine have unintended negative consequences. What is often lost in the methods employed by researchers when studying issues of representation (including one of the authors of this editorial)^14^,^15^ is that gender is too often considered through a binary lens. This can be a result of data limitations while performing a secondary analysis of large-scale databases, where sex is generally recorded in a binary manner, and gender may not be recorded at all. 2014 legislation supported updating electronic medical record systems to record gender in addition to sex, but this has not necessarily translated to data collection on physician gender makeup.^16^,^17^ Recent changes in application materials and reporting around gender by the AAMC^18^ and ACGME^19^ likely will allow for a more inclusive definition of gender in future studies.

Utilizing a binary lens can also be a consequence of studies designed toward advocating for equal representation and treatment of cis women where they are underrepresented. Dayal et al found that, despite being evaluated similarly as interns, over the course of residency, female emergency medicine residents were consistently evaluated lower than their male colleague across all subcompetencies.^20^ A similar difference was found in a national study of emergency medicine milestones,^1^ and the uniformity of this trend suggests implicit bias rather than diminished competency or skill, particularly considering that the study population began residency with similar skills and knowledge.^20^ Likewise in a study led by Mueller, female emergency medicine residents were more likely to get inconsistent feedback compared to their male colleagues, particularly surrounding culturally gendered attributes such as autonomy, independence, and assertiveness.^21^ While these studies indicate that female residents in emergency medicine are likely facing discrimination based on sex, there have not been studies that assess whether gender presentation plays a role in this discrimination, or if nonbinary and transgender trainees face additional discrimination related not only to sex, but also gender.

While the focus has long been on achieving gender parity between cisgender women and cisgender men in medicine, this is insufficient to support everyone. The consistent use of binary language and the exclusion of gender-diverse identities create gaps in our understanding of the treatment of gender-diverse individuals in our field. When reading the Gibney paper, we hope the reader considers how and by whom gender was assigned and how similar approaches in related research in education and workforce development can be reductive. An important consideration should be how, when trying to advocate for increased equity for one group, we may inadvertently create exclusionary language for others. In any case, repeatedly representing gender in binary terms has consequences for our understanding of the true make up of our emergency physician workforce, and how that representation may impact patient care. Continuing to focus on binary sex (male/female, to the exclusion of intersex people) and binary gender (men/women, to the exclusion of nonbinary and transgender people) will hamper efforts to create true equity for physicians of all identities in emergency medicine. In our efforts to address structural barriers for some historically underrepresented groups, such as cisgender women, we must not further discourage other underrepresented groups from considering emergency medicine.

## References

[1] Santen SA, Yamazaki K, Holmboe ES (2020). Comparison of male and female resident milestone assessments during emergency medicine residency training: a national study. Acad Med.

[2] Ross DA, Boatright D, Nunez-Smith M (2017). Differences in words used to describe racial and gender groups in Medical Student Performance Evaluations. PloS one.

[3] Greenwood BN, Carnahan S, Huang L (2018). Patient–physician gender concordance and increased mortality among female heart attack patients. Proc Natl Acad Sci.

[4] Cooper-Patrick L, Gallo JJ, Gonzales JJ (1999). Race, gender, and partnership in the patient-physician relationship. JAMA.

[5] Zink BJ (2005). Anyone, Anything, Anytime: A History of Emergency Medicine (Edition 1).

[6] Association of American Colleges (2017). More Women Than Men Enrolled in US Medical Schools in 2017.

[7] Association of American Colleges (2020). ERAS Statisitcs Association of American Medical Colleges.

[8] Association of American Medical Colleges (2016). Table B3. Number of Active Residents, by Type of Medical School, GME Specialty, and Gender.

[9] Sitkin NA, Pachankis JE (2016). Specialty choice among sexual and gender minorities in medicine: the role of specialty prestige, perceived inclusion, and medical school climate. LGBT Health.

[10] Legal L (2010). When health care isn’t caring: Lambda Legal’s survey of discrimination against LGBT people and people with HIV.

[11] Samuels EA, Tape C, Garber N (2018). “Sometimes you feel like the freak show”: a qualitative assessment of emergency care experiences among transgender and gender-nonconforming patients. Ann Emerg Med.

[12] Jetty A, Jabbarpour Y, Pollack J (2022). Patient-physician racial concordance associated with improved healthcare use and lower healthcare expenditures in minority populations. J Racial Ethn Health Disparities.

[13] Mansh M, Garcia G, Lunn MR (2015). From patients to providers: changing the culture in medicine toward sexual and gender minorities. Acad Med.

[14] Burkhardt J, DesJardins S, Gruppen L (2019). Diversity in emergency medicine: are we supporting a career interest in emergency medicine for Everyone?. Ann Emerg Med.

[15] Burkhardt J, DesJardins S, Gruppen L (2020). Identifying barriers to a diverse emergency physician workforce: identifying when and how leaks occur in the pipeline. Ann Emerg Med.

[16] Cahill SR, Baker K, Deutsch MB (2016). Inclusion of sexual orientation and gender identity in stage 3 meaningful use guidelines: a huge step forward for LGBT health. LGBT Health.

[17] Cahill S, Makadon HJ (2014). Sexual orientation and gender identity data collection update: US government takes steps to promote sexual orientation and gender identity data collection through meaningful use guidelines. LGBT Health.

[18] Association of American Colleges (2022). 2023 AMCAS Application Workbook.

[19] Accreditation Council for Graduate Medical Education (Academic Year 2020–2021) Data Resource Book.

[20] Dayal A, O’Connor DM, Qadri U (2017). Comparison of male vs female resident milestone evaluations by faculty during emergency medicine residency training. JAMA Intern Med.

[21] Mueller AS, Jenkins TM, Osborne M, Arora VM (2017). Gender differences in attending physicians’ feedback to residents: a qualitative analysis. J Grad Med Educ.

